# Perceived temporal distortions may explain the effect of precarity on intertemporal choices

**DOI:** 10.3389/fpsyg.2025.1671647

**Published:** 2025-12-05

**Authors:** Arjun Mitra, Nisheeth Srivastava

**Affiliations:** 1 Department of Cognitive Science, Indian Institute of Technology Kanpur, Kanpur, India; 2 Department of Computer Science and Engineering, Indian Institute of Technology Kanpur, Kanpur, India

**Keywords:** inter-temporal choices, time preference, delay discounting, unpredictability, subjective time, time distortions

## Abstract

Existing research on how environmental volatility affect preferences suggests that preference for delayed rewards reduces in the presence of adverse shocks. Researchers typically use reward discounting frameworks to explain these shifts in temporal preferences as shifts in discount rates. In this manuscript, we reinterpret these preference shifts as changes in perceived time duration. Using data from multiple studies, we found that decreases in choice probability for delayed rewards correspond to increases in perceived delay duration, while keeping discount rates constant. Our empirical study also showed that individuals who experienced extension of perceived time during volatility preferred short-term plans more. Based on these findings, we suggest the necessity of considering perceived temporal distortions to understand preference shifts when one encounters environmental volatility.

## 1 Introduction

Ever since Mischel's marshmallow test, time preference has been an attractive target for studies in cognitive psychology and behavioral economics (Mischel et al., 1989). Despite a proliferation of hypotheses and theories surrounding time preference, they share two common assumptions. One, people's proportion of preferring sooner choices, measured with multiple price lists, is nearly universally used to estimate a rate at which they discount the future (Andreoni and Sprenger, 2012). Two, this discounting rate is typically treated as a subject-specific trait, to be targeted as a dependent variable by downstream experimentation.

Some such investigations into economic conditions responsible for affecting time preferences have shown that variations in income and increased expenses lead to significant changes in intertemporal choices. Financial situations in households deteriorate significantly due to sudden increased expenses, especially for households who spend a substantial portion of their income on necessary goods like home energy and food (Cocco et al., 2016). Similar large-scale survey studies using household data have shown that households' propensity to consume increases immediately after encountering an unexpected, one-time expense that amounts to 10% of their income (Colarieti et al., 2024). These shock-based negative changes in preferences have also been found in the one-shot laboratory experiments where participants showed increased discounting when they faced an abrupt debit in their resources (Haushofer et al., 2013) or in within-subject, longitudinal studies where people faced an income loss due to the pandemic (Blain et al., 2021). Field studies done with Ethiopian farmers and Vietnamese households also show that people's discount rates are sensitive to income variations brought on by naturalistic shocks like rainfall shortfalls and droughts (Tanaka et al., 2010; Falco et al., 2019; Meles et al., 2024). The common observable across these studies suggest that unforeseen events like an unpredictable pay cut or an expense hike can lead people to think and act more present-focused, which underpins the role of expectation disruption on temporal preference shifts.

Standard discounted utility models pinpoint the reason for such future undervaluation as changes in the discount rate brought on by expectation-outcome mismatches. This is evident in exponential, hyperbolic (Laibson, 1997) or quasi-hyperbolic formulations (Mazur, 1984) where the utility of a delayed reward at time *T* is given by


$$\begin{array}{l} U\left(T\right)=\int_{t_{0}}^{T}f\left(t\right)u\left(t\right)dt \end{array}$$ (1)

and the discount function *f*(*t*) is given by


$$\begin{array}{l} \;\text{exponential discounting:}f\left(t\right)=e^{-kt}\\ \;\text{hyperbolic discounting:}f\left(t\right)=\frac{1}{1+kt}\\ \text{quasi-hyperbolic discounting:}f\left(t\right)=\beta \delta^{t}\;\forall \;t>0 \end{array}$$

In all such formulations, changes in the discount rate (or the present bias parameter) bring about changes in the perceived utility of delayed reward, however, the delay is considered to be invariant. On the contrary, extensive investigations in the time perception literature suggest that perceived time is a function of the amount of information processed in the judged interval (Thomas and Weaver, 1975) and expectation-outcome mismatches led by unpredictable stimuli can lead to distortions in duration judgments (Pariyadath and Eagleman, 2007). This suggests that delay distortion can be a possible psychological factor that changes the perceived utility of a delayed reward when one encounters volatility.

The effect of unpredictability on time perception is usually investigated using oddball paradigms in the perceptual literature. In these experiment, participants attend to a low probability, novel stimulus (*oddball*) in a sequence of high probability, repetitive stimuli and estimate the duration of the novel stimulus. The oddball is presented at varied durations (higher or lower than the repetitive stimuli), and participants note whether they perceive it to be longer or shorter than the standard stimuli. Across multiple studies, researchers have found that unpredictable stimuli are perceived to be longer than predictable ones (Tse et al., 2004; Ulrich et al., 2006; Pariyadath and Eagleman, 2007). These observations have been explained using an information processing account where unpredictable stimuli distort time by engaging attention and increasing the amount of information processed per objective time (Tse et al., 2004), or an arousal-based account where increased arousal speed up the internal clock causing longer perceived duration and better temporal discrimination than predictable stimuli (Ulrich et al., 2006).

Can subjective time affect our perceptions of the future? Probes into the psychological nature of time propose that people's perception of prospective duration is often non-linear and concave (Zauberman et al., 2009), and considering such logarithmic subjective time (instead of linear objective time) has shown to fit temporal choices better than standard exponential or hyperbolic ones (Takahashi et al., 2008). Similar theoretical investigations suggest that our internal biological clock may be stochastic and auto-correlated which can lead to different time estimations and time-inconsistent hyperbolic discounting for the same objective time (Ray and Bossaerts, 2011). Arguing on similar lines, (Cui 2011) demonstrated that a power law formulation of time perception also implies hyperbolic discounting.

Empirical investigation of these time-based models suggest that temporality is sufficiently sensitive to experimental manipulation and increased sensitivity to time can lead to increased discounting in the distant future (Ebert and Prelec, 2007). (Read 2001) found sub-additivity in time discounting and suggested subjective time perception as a possible mechanism for this effect. Furthermore, perceived increments in delay often lead to a switch in preference for the sooner reward, even if delayed rewards are preferred initially (McGuire and Kable, 2012) and individuals who overestimate delays tend to choose smaller, immediate rewards compared to people who underestimate them (Suo et al., 2014). Overall, these insights hint that people's perception of time may be incongruent with clock time and perceived delay distortions can lead to lowered preference for delayed rewards.

Now, deviations in choices brought on by unpredictable variations in income or expenses have been traditionally explained by shifts in discount rates. However, in the absence of objective measures of future discounting, standard discounting models assume some intrinsic utility of distal rewards, and subsequent changes in preferences are then explained by shifts in utility-based discount rates, offering a description rather than an explanation of the phenomenon. On the other hand, a delay distortion account indicates a possible psychological mediator connecting environmental precarity to intertemporal decisions.^1^ Unlike discount rates, delay distortions can be easily measured as deviations from an individual's sense of time or objective, clock time. Thus, a time distortion account opens up new scope for causal explanations of intertemporal preference changes without invoking shifts in discount rates.

Multiple studies across perceptual and decision literature suggest that—one, unpredictable stimuli distort duration judgments, two, environmental perturbations cause people to opt for sooner rewards, and three, perceived delay overestimations may lower future orientation. To our knowledge, no studies have attempted to theoretically or empirically connect these disjoint findings under one umbrella. In this manuscript, we attempt to do so by—first, developing a theoretical framework to identify if duration distortions can explain deviations in preferences, second, identifying lab and field studies investigating the effects of unpredictable shocks on intertemporal decisions and reinterpreting the observed shifts in discount rates as time distortions using our conceptual framework and third, empirically testing whether time distortions correlate with choice deviations.

## 2 Framework connecting choice deviations with time distortions

In this section, we develop a theoretical framework that derives estimates of temporal distortion from observed changes in choice probability of delayed rewards or documented shifts in discount rates. Imagine an individual's choice of delayed reward changes by Δ*p* as a result of an income or expense shock. If *p*_*b*_ and *p*_*a*_ denote the choice probability of delayed rewards before and after the shock, then the change in choice probability can be denoted by Δ*p* = *p*_*a*_−*p*_*b*_. Consider that we know the value of the sooner *r*_0_, the delayed reward *r*_*t*_, and its intervening delay *t*_*b*_.

Choice probabilities *p*_*b*_ and *p*_*a*_ can be represented by a softmax function as:


$$\begin{array}{l} p_{b}=\frac{exp\left(u_{b}\left(l\right)\right)}{exp\left(u_{b}\left(l\right)\right)+exp\left(u\left(s\right)\right)} \end{array}$$ (2)

$$\begin{array}{l} p_{a}=\frac{exp\left(u_{a}\left(l\right)\right)}{exp\left(u_{a}\left(l\right)\right)+exp\left(u\left(s\right)\right)} \end{array}$$ (3)

where *u*(*s*) denotes the utility of the sooner reward and is equal to its value *r*_0_. *u*_*b*_(*l*) and *u*_*a*_(*l*) denote the utility associated with the later reward before and after experiencing an income or expense shock.

Given we know the choice probability of delayed rewards prior to and post-shock, we can derive the utility associated with it from Equations 2, 3.


$$\begin{array}{l} u_{b}\left(l\right)=ln\left(\frac{p_{b}\times exp\left(u\left(s\right)\right)}{1-p_{b}}\right) \end{array}$$ (4)

$$\begin{array}{l} u_{a}\left(l\right)=ln\left(\frac{p_{a}\times exp\left(u\left(s\right)\right)}{1-p_{a}}\right) \end{array}$$ (5)

Assuming the delayed reward to be hyperbolically discounted over time, utilities of the reward *u*_*b*_(*l*) and *u*_*a*_(*l*) can be related to its actual value *r*_*t*_ using


$$\begin{array}{l} u_{b}\left(l\right)=\frac{r_{t}}{1+k_{b}\times t_{b}} \end{array}$$ (6)

$$\begin{array}{l} u_{a}\left(l\right)=\frac{r_{t}}{1+k_{a}\times t_{b}} \end{array}$$ (7)

where *k*_*b*_ and *k*_*a*_ denote the discount rates before and after the shock, and *t*_*b*_ denoted the time experienced before the shock. One can assume *t*_*b*_ to be equal to objective clock time or the individual's baseline subjective time.

Thus, using the previously obtained utility associated with the delayed reward before and after the shock, we can obtain *k*_*b*_ and *k*_*a*_ from Equations 6, 7 using


$$\begin{array}{l} k_{b}=\frac{1}{t_{b}}\times \left(\frac{r_{t}}{u_{b}\left(l\right)}-1\right) \end{array}$$ (8)

$$\begin{array}{l} k_{a}=\frac{1}{t_{b}}\times \left(\frac{r_{t}}{u_{a}\left(l\right)}-1\right) \end{array}$$ (9)

Thus, given values of delayed *r*_*t*_ and sooner reward *r*_0_, delay between them *t*_*b*_ and change in choice probability Δ*p* as a result of income or expense shock, one can determine the change in discount rate Δ*k* corresponding to Δ*p* using


$$\begin{array}{l} \Delta k=k_{a}-k_{b}=\frac{1}{t_{b}}\left[\left(\frac{r_{t}}{u_{a}\left(l\right)}\right)-\left(\frac{r_{t}}{u_{b}\left(l\right)}\right)\right]=\frac{r_{t}}{t_{b}}\left[\frac{1}{u_{a}\left(l\right)}-\frac{1}{u_{b}\left(l\right)}\right] \end{array}$$ (10)

Traditionally, researchers have studied changes in choice probability brought on by lab-induced instability or environmental perturbation in the framework of the discount rate. However, we aim to identify changes in perceived time that can correspond to reported changes in choice probabilities. As noted above, temporal distortions can be calculated as the difference between perceived time during experienced volatility and at baseline state or between subjective and clock time. Thus, a positive deviation would imply subjective time to be longer than baseline subjective time or objective clock time (time dilation), and a negative deviation would imply perceived time to be shorter (time contraction).

For this exercise, we assume that distortions in time bring about changes in choice probability instead of shifts in the discount rate. Therefore, *u*_*b*_(*l*) and *u*_*a*_(*l*), as mentioned in Equations 6, 7, would be noted by


$$\begin{array}{l} u_{b}\left(l\right)=\frac{r_{t}}{1+k_{b}\times t_{b}} \end{array}$$ (11)

$$\begin{array}{l} u_{a}\left(l\right)=\frac{r_{t}}{1+k_{b}\times t_{a}} \end{array}$$ (12)

*t*_*b*_ and *t*_*a*_ represents the perceived delay duration before and after shocks. Note that Equation 12 is similar to Equation 11, except that we consider changes in perceived delay to bring about changes in utility post-shock while keeping the discount rate constant.

One can obtain *t*_*b*_ and *t*_*a*_ using *u*_*b*_(*l*) and *u*_*a*_(*l*) from Equations 11, 12.


$$\begin{array}{l} t_{b}=\frac{1}{k_{b}}\times \left(\frac{r_{t}}{u_{b}\left(l\right)}-1\right) \end{array}$$ (13)

$$\begin{array}{l} t_{a}=\frac{1}{k_{b}}\times \left(\frac{r_{t}}{u_{a}\left(l\right)}-1\right) \end{array}$$ (14)

Assuming discount rates to be unchanged due to shocks, one can derive distortions in perceived time Δ*t* using


$$\begin{array}{l} \Delta t=t_{a}-t_{b}=\frac{1}{k_{b}}\left[\left(\frac{r_{t}}{u_{a}\left(l\right)}\right)-\left(\frac{r_{t}}{u_{b}\left(l\right)}\right)\right]=\frac{r_{t}}{k_{b}}\left[\frac{1}{u_{a}\left(l\right)}-\frac{1}{u_{b}\left(l\right)}\right] \end{array}$$ (15)

Using Equations 10 and 15, one can equate temporal distortions with shifts in discount rate using


$$\begin{array}{l} \Delta t=\frac{t_{b}}{k_{b}}\times \Delta k \end{array}$$ (16)

Therefore, shifts in choice probability can be interpreted as delay distortions instead of changes in discount rate using Equation 16. This equation tells us that shifts in discount rate scaled by an individual's discount rate before experiencing shocks would equal delay distortions scaled by the perceived delay between rewards before shocks. This also tells us that positive and negative changes in discount rates should correspond to time dilation and contraction in the context of unpredictable volatility.

## 3 Reinterpreting preference shifts as time distortions

Utilizing the conceptual framework, we investigate the order of time distortions corresponding to choice probability changes documented in lab and field studies. In these studies, variations in income or expenses are often formulated as narratives depicting a sudden loss in income (Bickel et al., 2016), as abrupt resource debits on an effortful task in the lab (Haushofer et al., 2013), or studied in the field as unpredictable environmental perturbations like rainfall anomalies (Falco et al., 2019). Given empirical reports of negative preference shifts caused by unpredictable shocks (shown in Table 1), we use our framework to derive the time distortions corresponding to such preference deviations.

**Table 1:** Summary of studies examining the impact of negative income or expense shocks on temporal choices.

**Study**	**Sample**	**Observed effects**	**Strength of effect**	**Scaled time distortions**
1. (Haushofer et al. 2013)	148 students (lab)	Hypothetical income shocks deducted money in a computerized task. The high-endowment group with a negative shock showed greater discounting than the low-endowment, no-shock group.	Negative income shock group showed lower indifference points than the poorly endowed ones (*d* = -0.46).	med = 0.539 95% CI (0.469, 0.61)
2. (Bickel et al. 2016)	599 individuals (MTurk)	Participants received hypothetical narratives of varied valence. Abrupt, negative income narratives increased discount rates more than neutral ones in both future gain and loss domains.	Δln(*k*) = 1.29 for a future gain scenario with a negative income shock vs. neutral narrative.	med = 3.37 95% CI (1.69, 6.08)
3. (Falco et al. 2019)	1,160 farmers in Ethiopia	Higher rainfall anomalies led to increased income variation, resulting in higher subjective discount rates.	Increased rainfall anomalies reduced farm income (α = 0.66 ± 0.33), leading to increases in discount rate (β = −0.05 ± 0.02).	med = 0.326 95% CI (0.288, 0.366)
4. (Blain et al. 2021)	184 individuals (longitudinal data)	Income shocks, measured via subjective ratings, during the COVID-19 market crash (March–June 2020), increased preference for sooner rewards.	Discount rates increased with every unit increase in perceived shocks discount rates (β = 0.14 ± 0.06).	med = 0.158 95% CI (0.135, 0.181)
5. (Meles et al. 2024)	5,600 rural households in Ethiopia	Income shocks measured using rainfall deviations and self-reported droughts over two years. Impatience increased as a result of both empirical (rainfall shortfalls) and self-reported (drought) shocks.	Preference for later rewards decreased as a function of drought incidence (β = −0.14 ± 0.05) and prior-year rainfall shortages (β = −0.26 ± 0.07).	Droughts: med = 0.064 95% CI (0.056, 0.075); Rainfall: med = 0.150 95% CI (0.135, 0.166)

The different columns denote the sample characteristics, observed effects and strength of the observed effects in terms of changes in discount rates. The final column shows the estimated time distortions (median and 95% CI) derived from observed changes in discount rates using our theoretical framework.

Our primary criterion of choosing these articles was to interpret literature published within the last ten years that were conducted in diverse contexts using varied samples and employed diverse methods of shock formulation. Thus, we selected these five studies, which were conducted either in the lab or in the field, using different socio-economic samples, and examined real-life shocks (rainfall anomalies or droughts) or simulated ones.

Using data from studies mentioned in Table 1, we extract choice probability shifts or changes in discount rates induced by income or expense shocks. For studies that report Δ*p*, we derive Δ*k* using *p*_*b*_ and *p*_*a*_, and the procedure mentioned in the previous section. Next, using these Δ*k* (difference in mean discount rates after and before shocks), we calculated scaled discount rate shifts Δ*k*/*k*_*b*_ which gave us the scaled time distortions Δ*t*/*t*_*b*_ as given by Equation 16. For these computations, we considered subjective time at baseline *t*_*b*_ to be equal to objective clock time, since we did not have individuals' time estimates. Finally, we constructed a distribution of such scaled time distortions by bootstrapping 10,000 samples from each dataset which gave us the median time distortion and its 95% confidence interval for each study (i.e., the 50th, 2.5th, and 97.5th percentile respectively).

**Figure 1:**
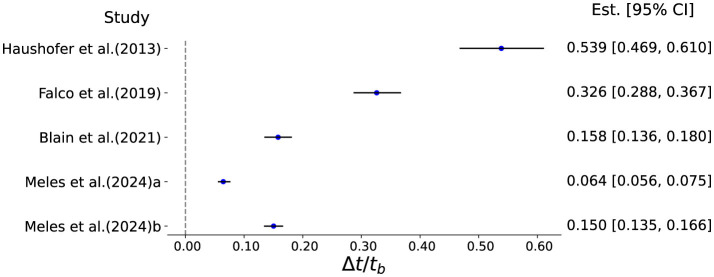
This figure shows the median scaled time distortions (Δ*t*/*t*_*b*_) along with its confidence intervals calculated from shifts in preference for studies reported in Table 1. The zero line depicts null time distortion i.e., subjective time = objective time. Meles et al. (2024) a and b refer to the effects of droughts and rainfall shortfalls on shifts in preference separately.

As shown in Figure 1, we find that median scaled time distortions lie in the range of 0.064 to 0.539 for all studies except Bickel et al. (2016)'s study, which shows a median time distortion of 3.37 with its 95% CI (1.69, 6.08). Thus, for a 1,000 ms delay in future rewards, the median time distortions range between 64 ms and 539 ms across studies. Usually, in perceptual studies, unpredictable stimuli are documented to cause temporal distortions of magnitudes from 5% (Ulrich et al., 2006), 12% (Pariyadath and Eagleman, 2007) to as high as 35% (Tse et al., 2004). Thus, for a 1,000 ms standard duration, the estimated time distortions can vary in the range of 50–350 ms for perceptual studies, which seems to be reasonably aligned with our derived duration distortions.

We also find that time distortions show considerable heterogeneity across studies, which may have resulted from how income or expense shocks were operationalized in each experiment. While some have investigated income shock as abrupt hypothetical monetary debits (Haushofer et al., 2013), others have measured income shock subjectively (Blain et al., 2021) or using narrative depicting consequential adversity (Bickel et al., 2016). In (Bickel et al. 2016)'s study, the negative income shock narrative is framed broadly as a sudden job loss with being evicted and unqualified for unemployment. Thus, the observed inflated time distortion may be a result of this broad framing encompassing detailed negative consequences of adversity. Overall, these results indicate increased temporal distortions can lead to negative shifts in preference if one considers discount rates to be invariant within individuals.

## 4 Effects of lab-induced precarity on intertemporal choices

In this section, we investigate whether temporal distortions are related to preference shifts using our lab experiments. To this effect, we calculated time distortions from deviations in choice probability using data from our previously published experiment, and also conduct a replication experiment with explicit felt time probes and a larger sample size to check whether these results hold empirically.

### 4.1 Preference shifts and time distortions in original study

In previously published experiments [unpredictable arm of experiment 3 in Mitra et al. (2025)], we had simulated periods of stability and volatility using a computer game. Participants earned money by growing and harvesting crops while navigating stable and turbulent farming conditions. During stability, participants saw minimal farming expenses, land rents, and crop losses. However, during precarious conditions, farming expenses and crop losses would unpredictably increase, resulting in an unforeseen drop in their income.

Participants chose from a fixed array of three crops (apple, rice, and teak), which systematically varied in their growth time (long-term and short-term) and profitability (risky and non-risky). Short-term crops that could be harvested at the end of each trial, and the long-term crop matured after six trials. Risky crops were designed to provide high profit during stability and high loss (or low profit) during volatility. Thus, we had three predefined crops: non-risky long-term teak, non-risky short-term rice, and risky short-term apple. We purposefully made an unbalanced design so that people could choose a single, safe, long-term option.

The experiment started with a practice block, followed by low volatility (LV) and high volatility (HV) blocks. Specifically, the game consisted of 96 trials, of which the first 24 trials were practice rounds, followed by 24 trials depicting stability, and ended with 48 trials depicting precarity. Out of these 48 trials, each participant faced 16 expense shocks during which they faced significant farming expenses, high crop losses, and land rent (for details, check out Section 6.1).

Our goal was to check whether individuals' crop plantation strategy shifted from the long-term to short-term as a function of experienced precarity. Thus, for each individual, switches in crop production were calculated by estimating the difference in the number of apples, rice, or teak planted before and after shock, which was averaged over all encountered shocks (for details, see Section 6.2). We found that preference for the long-term crop teak decreased, while the preference for short-term rice increased.

Now, what may be the reason for participants to choose the safe option (rice) over the risky choice (apple) when moving away from teak? Teak was designed to be the optimal or profit-maximizing crop option when the environment was stable. However, as shocks became more recurrent, opting for the long-term crop during volatile periods led to faster capital depletion. Among the short-term crops, apples were considered the riskier choice, and therefore choosing rice yielded a higher profit than apples during volatile periods. Thus, opting for the safe option (rice) was the optimal choice when shocks are incessant. Participants inductively determined the optimal choice in precarious conditions by selecting rice when shifting away from teak (for a detailed calculation of optimal crop choice, please see Mitra et al., 2025).

As noted in Section 2, one can derive these shifts in long-term preference as changes in discount rate. However, if one assumes discount rates to be subject-specific and invariant, changes in choice probabilities can also be construed as distortions in time. Thus, using our conceptual framework, we interpreted the observed preference deviations as shifts in discount rates and also reinterpreted them as time distortions. Utilizing these results, we checked whether delay distortions can be reliably estimated from these preference shifts using our theoretical framework.

For our current purpose, we derived the probability of choosing long-term teak before *p*_*b*_ and after *p*_*a*_ encountering shocks by dividing the total number of teak planted by the total number of crops chosen (teak + apple + rice). Using *p*_*b*_ and *p*_*a*_, we obtained the utility of choosing teak before *u*_*b*_ and after *u*_*a*_ shocks (using Equations 4, 5). *u*_*b*_ led us to individuals' measures of discount rate pre shock *k*_*b*_ (using Equation 8) which led us to the pre and post-shock time estimates *t*_*b*_ and *t*_*a*_ corresponding to observed preference shifts *p*_*b*_ and *p*_*a*_ (using Equations 13, 14). Finally, these time estimates gave us our scaled time distortions Δ*t*/*t*_*b*_. ^2^

While time distortions can be measured as deviations from clock time or deviations from individuals' baseline subjective time, for this analysis we calculated time distortions from objective time (i.e., *t*_*b*_ = *t*_*o*_) since we did not have participants' time estimates. In Mitra et al. (2025), we had excluded two participants based on an 1.75*IQR exclusion criteria and obtained a sample size of 28 individuals. In this analysis. we also excluded one more individual who showed an extreme scaled discount rate distortion of –6.65 leading to a sample size *n* = 27.

**Figure 2:**
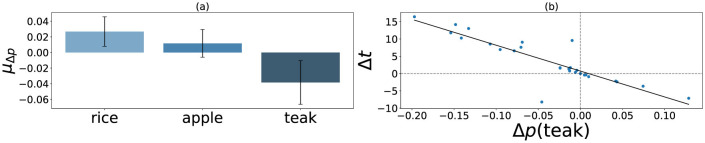
Figure **(a)** shows the mean change in choice probability (μ_Δ*p*_) across crops as a result of lab-induced environmental precarity (*n* = 27). Error bars represent 95% CI. Figure **(b)** shows that the decreases in observed teak probability [Δ*p*(teak)] are associated with increases in time distortions (Δ*t*) estimated from the data (*r* = –0.87, *p* < 0.001).

Figure 2a shows the mean choice probability deviations calculated as a function of shocks. Similar to our previous results, preference for the long-term crop teak decreased significantly after encountering shocks [*M* = –0.04, *SD* = 0.07, *t*_(27)_ = –2.83, *p* = 0.004, *d* = –0.54]. Among the short-term crops, people preferred the non-risky choice option rice post shock [*M* = 0.03, *SD* = 0.05, *t*_(27)_ = 2.99, *p* = 0.006, *d* = 0.57]. At the same time, no significant change was observed for the risky crop apple [*M* = 0.01, *SD* = 0.05, *t*_(27)_ = 1.14, *p* = 0.26, *d* = 0.22]. In our experiment, a lowered teak preference would consequently cause an increased preference for short-term crops, as no field plots could be left unplanted by design. Thus, along with decreased preference for the long-term crop, we found that participants preferred the low-risk short-term option rice more compared to the high-risk choice apple.

Section 2 tells us that scaled distortions in time Δ*t*/*t*_*b*_ should be equal to scaled distortions in discount rates Δ*k*/*k*_*b*_, and we found that to be the case with this data (*M* = 0.24, *SD* = 0.42). Each trial in our experiment was designed to last for 15 s, including trials on which participants experienced shocks. Thus, using Equation 16, we calculated the estimated time distortions Δ*t* using objective time *t*_*b*_ = *t*_*o*_ = 15s. Δ*t* showed a right-skewed distribution with *m* = 3.6, *med* = 1.6, and *sd* = 6.35, suggesting a perceived time expansion at the group level. More importantly, a correlation between Δ*p* and Δ*t* (Figure 2b) suggests a strong negative correlation of –0.87 (*p* < 0.001), indicating a potential association of time dilation with negative preference deviations at the individual level.

### 4.2 Preference shifts and time distortions in replication study

Insights from lab and field studies (including our experiments) suggest a link between subjective time and present-focused behavior. However, whether subjective experiences of time expansion are actually associated with preference shifts has been left unresolved. In an attempt to answer the question, we ran a replication of the previous experiment with a larger sample size, where we asked individuals to denote their felt time estimates at different junctures in the game. Our main goal was to see whether participants shift away from long-term options as before and whether these preference shifts correlate with felt time distortions.

Our overall design was similar to the previous one, where participants planted and harvested crops (apple, rice, and teak) to maximize their capital over time while navigating stable and volatile farming environments. However, we made two key changes in the current design:

- Stability post-volatility: In the previous experiment, the farming game ended with participants encountering 48 trials depicting volatility. Some participants had remarked that they did not choose teak in the last six trials as they would not be harvestable by the time the game ends. This led to the exclusion of shock trials occurring in the last six trials from further analysis. In the current design, we added 12 trials of stable farming conditions to mitigate that effect. Thus, our experiment started with 24 trials of practice rounds, followed by 24 trials of stable farming conditions, then 48 trials of volatility, and ended with 12 trials of stability, increasing the total number of trials from 96 to 108.
- Felt time measure: In the current design, we asked participants to denote the felt duration of the previous trial using a slider ranging from 1s to 60s at different points in the game. Each participant answered this question nine times out of 108 trials—thrice during the first 24 trials of stability and six times during the following 48 turbulent trials. Within these 48 trials, we asked about felt time thrice immediately after a resource shock and thrice when there were no shocks.

The felt-time probes were placed so that each participant faced these questions roughly at the beginning, middle, and end of the stable and volatile periods. For example, participants answered about their felt time during trials 25, 34, and 45 in the stable period, which lasted from trials 24–48. This pseudo-random design enabled us to get subjective time measures over the entire streamlined and turbulent period while making it impossible for participants to guess when the next probe would appear. Furthermore, we probed subjective time every 8–12 trials to prevent participants from anchoring on their previous estimates. Time probes were placed such that participants denoted their felt time immediately after the end of the ongoing trial and before planting or harvesting crops for the subsequent trial.

**Figure 3:**
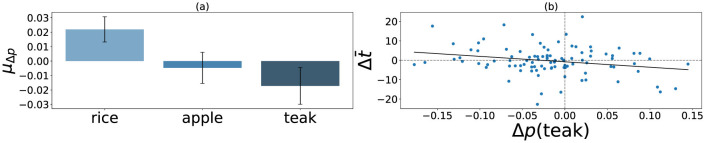
Figure **(a)** shows the mean change in probability of crop choice (μ_Δ*p*_) as a result of lab-induced environmental precarity (*n* = 96). Error bars represent 95% CI. Figure **(b)** shows that perceived distortions in time from individual's own baseline ($\Delta \bar{t}$) is negatively correlated to shifts in probability of choosing the long-term crop teak (Δ*p*(teak)) (*r* = −0.24, *p* = 0.008).

Our analysis protocol for this experiment was similar to the one used in Mitra et al. (2025). For each individual, we aimed to check whether individuals' crop plantation strategy shifted from the long-term to short-term as a function of experienced precarity. Thus, we calculated switches in crop production by estimating the probability of choosing apples, rice, or teak before and after shock, and averaging them over all encountered shocks. Using a 1.75*IQR exclusion criterion, we identified six participants as outliers who were excluded from further analysis. As expected, we found that probability of choosing teak significantly decreased post shock across our sample of 96 individuals [*M* = –0.02, *SD* = 0.054, *t*_(95)_ = –2.63, *p* = 0.005, *d* = –0.27] as shown in Figure 3a. Furthermore, among the short-term crops, rice preference increased [*M* = 0.022, *SD* = 0.044, *t*_(95)_ = 4.9, *p* < 0.001, *d* = 0.50] and apple preference showed no change [*M* = –0.005, *SD* = 0.054, *t*_(95)_ = –0.85, *p* = 0.396, *d* = 0.087] as a result of experienced shocks. Thus, participants again preferred the low-risk short-term choice rice more than high-risk apple when shifting their preference away from long-term option teak.

Now, instead of estimating felt time deviations using our conceptual framework, we calculated time distortions from the participants' data. As noted before, participants reported their subjective trial duration thrice during the stable period, thrice during non-shock turbulence, and thrice during shock-riddled turbulent trials. The average of these time reports constituted the baseline (*t*_*bs*_), non-shock turbulent (*t*_*nst*_), and shock turbulent (*t*_*st*_) subjective time. Since each trial lasted for 15s, we also had a measure of objective, clock time *t*_*o*_ for reference. Thus, time distortions were calculated in three ways:

- Changes from baseline condition: $\Delta \bar{t}$ = *t*_*st*_−*t*_*bs*_
- Changes in felt time within turbulence: $\Delta \overset{´}{t}$ = *t*_*st*_−*t*_*nst*_
- Changes in time during turbulence with respect to objective time: $\Delta \hat{t}$ = *t*_*st*_−*t*_*o*_

The distribution details of all three estimates of time distortions are reported in Table 2. We found felt time distortions from individuals' baseline $\Delta \bar{t}$ and within turbulence $\Delta \overset{´}{t}$ to be normally distributed. The distributions showed a minor underestimation of perceived time (since mean and median < 0), which was not statistically significant. On the other hand, felt time distortions from objective time $\Delta \hat{t}$ showed a right-skewed distribution (mean > median) with statistically significant evidence for time overestimation [*t*_(95)_ = 2.7, *p* = 0.008, *d* = 0.27, 95% CI (0.88, 5.8)]. Interestingly, the average distortions $m_{\Delta \hat{t}}=3.34$ closely match the estimated mean time distortion in the previous study *m*_Δ*t*_ = 3.6. These results suggest that precarious conditions expanded subjective time relative to objective time but not from individuals' baseline perceptions at the group level.

**Table 2:** Summary statistics of time distortion distributions and their correlation with shifts in long-term teak preference.

**Time distortions**	** $\Delta \bar{t}$ **	** $\Delta \overset{´}{t}$ **	** $\Delta \hat{t}$ **
Mean	–0.32	–0.11	3.34
Median	–0.295	–0.19	–0.39
SD	7.33	5.02	12.09
Corr with Δ*p*(teak)	–0.24 (0.008)	–0.14 (0.09)	–0.11 (0.13)

All correlations are Pearson's *r*, and bracketed values denote p-values.

Next, we ran correlations between shifts in teak preference and felt time distortions. Applying a Bonferroni correction for three comparisons, we found that shifts in probability of choosing teak Δ*p*(teak) were significantly correlated with felt time distortions from individuals' baseline $\Delta \bar{t}$, as noted in Table 2 and Figure 3b. Subjective time distortions within turbulence $\Delta \overset{´}{t}$ and from objective time $\Delta \hat{t}$ were also negatively correlated to teak preference shifts, but not statistically significant. Overall, at the individual level, participants who reported expansion of delay durations during volatility compared to stable conditions were less likely to prefer long-term crops in the face of adversity. Individuals also reported time extension relative to clock time, and we found the effect of these time distortions on long-term plans to be small but directionally consistent.

As found in the original study, we again observe that people tend to shift to the non-risky short-term option (rice) when moving away from the long-term choice (teak) in the replication study. We also tested whether reported time distortions were related to these risk preferences and found no significant correlations between changes in preference for the safe short-term crop option and perceived deviations in time. This leads us to believe that choosing the safe, short-term option was a resource conservation strategy employed by individuals under incessant shocks, and thus people acted as resource-bounded optimizers in line with our optimality estimations.

Overall, insights from this experiment reaffirm our previous intuition. Our conceptual framework, developed in Section 2, suggested that deviations in long-term preference can be construed as shifts in subjective time if one considers discount rates to be invariant. Across studies mentioned in Section 3 and our previous experiment, we found that perceived temporal extension may contribute to increased short-term preference. Results from this experiment provide the empirical validation that people perceive time differently during stability and turbulence and these perceptual distortions are closely linked to their intertemporal decisions. Since, all individuals in this experiment encountered the same number and magnitude of resource shocks, the variation in preference shifts can be attributed to their sense of time.

## 5 Discussion

This work set out to examine whether shifts in preference induced by unpredictable shocks can be interpreted as shifts in subjective time. The novelty of our approach lies in unifying unpredictability, temporal distortions, and discounting under one overarching proposition, which enables us to understand how observed changes in the environment may cause a psychological drift that can lead to changes in decisions. While traditional theories have interpreted such preference shifts as shifts in discount rates (Haushofer et al., 2013; Meles et al., 2024), we suggest an alternative theory: that short-term preferences may arise from how time is perceived under precarity. Across multiple studies, we observe that time distortions reported in decision-making literature are of comparable magnitude to those reported in timing studies. Using our lab experiments, we reinterpreted discount rate shifts as distortions in felt time and verified that time distortions can account for long-term preference shifts induced by precarity.

Although subjective time estimates during volatile conditions increased compared to objective time in the replication study, we found a slight average underestimation from individuals' own perceptual baseline. Now, increased attention to an ongoing task (diverted from time) or increased working memory load is often known to shorten perceived time (Polti et al., 2018). Unpredictable exogenous shocks in our experiment may have played a similar role by diverting increased attention to ongoing turbulence, leading to the observed underestimation. Furthermore, around one-third of our sample shows a negative time distortion and a negative shift in teak preference. This suggests that the influence of shocks on preferences may not be completely mediated by perceived time. Unpredictable perturbations can induce liquidity constraints on individuals, leading to an increased preference for short-term plans. Other cognitive mechanisms, such as increased attentional tunneling or cognitive load, may also be at play here.

Overall, our theoretical framework and empirical results lie in accordance with investigations that highlight the need to move beyond standard explanation under the discounting framework and consider the role of time perception in intertemporal choices. Subjective time need not be equivalent to objective, clock time, and distortions in time can modulate people's preferences (Zauberman et al., 2009; Bradford et al., 2019). Studies also suggests that perceived time is often non-linear and logarithmic in nature (Brocas et al., 2018). Thus, incorporating a Weber-Fechner formulation of subjective time instead of clock time leads researchers to suggest that lack of self-control and time-inconsistent hyperbolic discounting may be understood in light of this non-linear time perception (Takahashi et al., 2008; Zauberman et al., 2009; Cui, 2011; Takahashi, 2016).

These ideas are also supported by insights from timing studies which suggest that our perception of time is highly malleable to our lived experiences. Subjective time is often a function of attentional demands (Tse et al., 2004), information processing (Thomas and Weaver, 1975), or predictability of the stimuli (Pariyadath and Eagleman, 2007). In our context, environmental volatility may demand excessive attention to immediate troubles or impose the need to exercise high cognitive effort (Mani et al., 2013's attentional tunneling and high load). Such contextual factors may warp individuals' perception of time and how the future is visualized. Thus, environmental instability can affect mental time, affecting how far into the future one can afford to plan.

Indeed, the influence of subjective time on decisions can explain why people may prefer delayed rewards initially and defer them later, also known as preference reversals (Thaler, 1981). McGuire and Kable (2012) demonstrated that when the delay in intertemporal choices seems to increase over time (waiting for a phone call), people often prefer the delayed rewards initially and forgo them later. Indeed, some have suggested that the formulation of time-inconsistent, hyperbolic discounting stems from mischaracterizing subjective time as objective time in intertemporal decisions (Bradford and Doucette, 2023). Thus, a duration distortion account explains present-focused behavior by recognizing a subjective expansion or dilation of time, leading one to defer waiting, even though waiting is preferred initially.

The idea of incorporating time distortion in discounting models has been introduced previously using time-scaled discount factors, where the time-scaling factor represents speeding up and slowing down of time (Read, 2001; Andersen et al., 2014). Time scaling makes similar predictions to our work—if the delay to a reward delivery seems to be extending, one will exhibit higher discounting, and hence, one may choose to forego the delayed reward. Empirical validations of such a phenomenon have been recently observed in studies that indicate how one experiences and estimates time can predict future discounting in the lab (Chen and Zhao, 2024) and impulsive behaviors like snacking and over-eating in the real world (Isham et al., 2022).

Now, adopting a temporal distortion account of preference shifts offers several benefits. For example, it avoids reliance on utility-based assumptions, which are difficult to apply to abstract goals like buying a house or landing a job. Second, it offers a natural reference point (for example, subjects' own baseline subjective time or clock time), allowing deviations to be measured, unlike discount rate accounts, which lack an objective measure (a “true zero” point). Furthermore, standard economic theories often assume time preferences to be “pure” and non-mutable and different from time discounting (Frederick et al., 2002). However, studies investigating the effect of shocks on preference have questioned this assumption, as discount rates increase proportionally with environmental perturbations. A time distortion perspective accommodates these findings by offering an alternative explanation where preference changes are caused by how time is perceived, keeping internal time preferences unperturbed.

An implicit assumption built into our theory is that individual's discount rates are constant. In addition to the formulation and measurement difficulties associated with discount rates, we think sensitivity to delayed outcomes may be a stable and pervasive individual characteristic as often demonstrated by studies which show positive correlations for discounting across domains and outcomes (Odum, 2011). Thus, we propose that state-level changes in discount rates derived from observed shifts in preference can be attributed to subjective mental states such as time.

Now, we think the temporal distortion framework depends on the time scale of the decision, which can be reliably estimated. This includes durations that can be mentally simulated or perceived as a delay (from days or weeks to a few years). When the delay becomes extremely short (e.g., one hour) or extremely long (e.g., a decade), preferences are likely to be dominated by other cognitive mechanisms in addition to temporal distortion. For very short delays, subjective time is nearly veridical, and changes in preference are more likely driven by arousal or attention-based mechanisms. On the other hand, for very long delays, we think people would rely on semantic representations of time (such as “a long time from now”) rather than continuous temporal simulation. In such cases, future valuation may be influenced by abstract beliefs about uncertainty or affective responses to instability rather than distortions in perceived duration.

While our farming game simulated the lived experience of individuals in low socio-economic conditions, our sample characteristics are limited to college students who come from relatively affluent families. To investigate the role of perceived time at the intersection of volatility and preferences in low socio-economic conditions, future work should aim to replicate this study with a more appropriate sample. Furthermore, since we simulated the lived experiences of being in dire situations, we investigated only the effect of adverse shocks on intertemporal choices. We believe that positive shocks, such as a sudden bonus or income, should increase preference for delayed rewards. Consequently, individuals who experience time dilation should act more present-biased compared to those who experience time contraction.

In conclusion, we propose that a duration distortion account can explain preference changes without invoking latent constructs like impatience or inability to delay gratification in the face of instability. A duration distortion account also explains why individuals in low socio-economic conditions persistently discount the future. Uncontrollable mortality risks (Pepper and Nettle, 2017) or pessimistic bias toward the future (Das et al., 2020) prevalent in low socio-economic individuals can be understood in the light of time distortions—if the future looks too distant to be true, one would naturally invest in the present. Taken together, our findings underscore the need to understand intertemporal discounting as a dynamic interplay of instability, time, and decisions.

## 6 Methods

Our motivation was to create a close-to-real-life platform where people would experience an intermittently unstable financial environment while planning and choosing between short-term and long-term options. Therefore, we designed our experimental paradigm as a simulator game where participants played the role of a farmer who planted and harvested crops over multiple trials, while observing farming expenses. To depict environmental precarity, we designed the farming expenses to randomly go overboard on some trials and we check how long-term preference changed as a function of unpredictable shocks.

### 6.1 Farming game design

The experiment started with a practice block followed by blocks of low volatility (LV) and high volatility (HV). The key difference in the current study and the Mitra et al. (2025)'s Experiment 3 is the placement of these blocks:

- Original study:
  Practice rounds (24 trials) → LV block (24 trials) → HV block (48 trials)
- Current replication study:
  Practice rounds (24 trials) → LV block (24 trials) → HV block (48 trials) → LV block (12 trials)

Since, the game ended with the HV block in the original study, individuals noted that they were not motivated to plant teak in the last six trials primarily because they would not be reapable before the game ends. To eliminate this confound, we added a low volatility block at the end of the game in the current experiment.

#### 6.1.1 Unforeseen expenses as precarity

**Figure 4:**
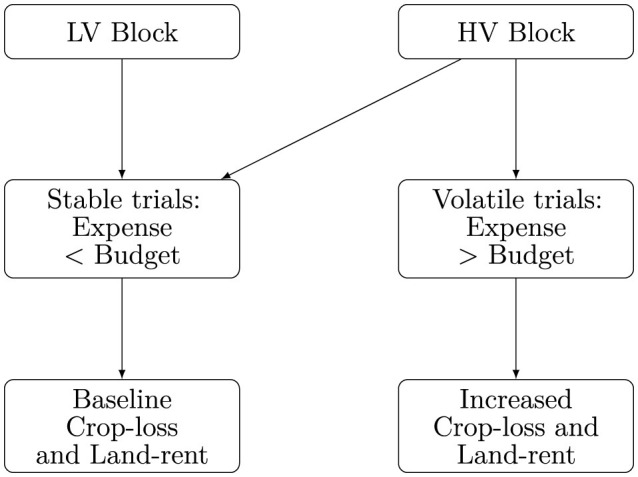
Flowchart showing experimental manipulations of our farming environment. Participants encountered an LV block followed by an HV block. The LV block was designed to be stable with farming expenses less than the stipulated budget. HV block, however, had several volatile trials interspersed among stable trials where participants encountered a sudden increase in farming expenses and crop losses.

Now, the question is how should low and high volatility be operationalized? We often allocate budgets to various accounts to manage daily expenses, such as having a monthly household bill account. Precarity happens when such accounts are depleted due to unforeseen, significant expenses. For instance, one may receive a substantial medical bill that eats up their household expenses budget. With this intuition, we designed our game's stable and volatile farming conditions. Figure 4 briefly summarizes these manipulations.

Volatile farming conditions (HV trials) were marked by unpredictable resource demands such that farming expenses exceeded a stipulated budget. During stable (LV trials), these farming inputs were always designed to be less than the budget, signaling a non-volatile environment. Precisely, we allocated a budget *B* = 5, 000 from which farming expenses were debited on each trial. Farming expenditures were sampled from a normal distribution [*N*(3750, 760)for LV, and*N*(3750, 2845)for HV blocks]. If the sampled value was greater than *B*, participants saw increased crop losses and land rents. This was our independent variable i.e., our depiction of precarity simulated in the virtual environment.

Now, crop losses on each trial were sampled from a binomial distribution *B*(*n, p*) where *n* denoted the number of crops and *p* the probability of crop loss. We designed the game such that participants would see baseline losses (30% of apples, 20% of teak, and 20% of rice) during stable LV trials and increased crop loss (80% of apples, 50% of teak, and 50% of rice) during volatile HV trials (noted as Loss Factor in Table 3). Similarly, land rent was also designed to increase from a baseline of 200 to 1, 000 coins on these trials. Thus, in these shock trials, participants would experience turbulence in the form of budgetary depletion caused by increased farming expenditures, resulting in increased crop losses and expenses.

**Table 3:** This table shows the different types of crops, their growth time, and buying and selling prices.

**Crops**			
**Type**	**Apple**	**Rice**	**Teak**
Yield time	1 trial	1 trial	6 trials
Buying price	50 coins	50 coins	50 coins
Selling price	350 coins	275 coins	1,700 coins
Loss factor	0.3 (0.8)	0.2 (0.5)	0.2 (0.5)

The loss factor indicates that the probability of crop loss obtained using a binomial distribution during periods of stability and instability (values in brackets).

#### 6.1.2 Crop choices as intertemporal investments

On each trial, participants sowed their crops of choice, observed various farming inputs and crop expenses, and finally harvested their chosen crops at the end of the trial. Crop choices consisted of apples, rice, and teak, where apples and rice were the short-term crops (harvestable in one trial) and teak was the long-term option (harvestable in six trials).

Apples and rice were designed as the sooner, immediate reward because they took minimal time to grow (yield time of 1 trial) and gave smaller earnings upon harvesting (selling—buying price). On the other hand, teak was our depiction of the delayed, larger reward as it had the longest yield time of 6 trials with yields greater than our short-term options. Thus, crop varieties were our depiction of intertemporal choices, and we were interested in finding whether people shift from the long-term crop to short-term ones under the influence of volatile conditions.

While teak was our low-risk, long-term crop choice, we varied the riskiness of our short-term crops such that apple was our high-risk, high-return option, and rice was the safe, low-return choice. Thus, apples were designed to provide higher profit than rice in low-volatility conditions, but instability would amplify the loss of apples, leading to lower profits than rice. For example, choosing 10 apples and 10 rice would lead to a loss of eight apples compared to five rice in periods of instability (see Loss Factor in Table 3). We varied the riskiness of the short-term crops as we were interested in what participants would opt for when switching away from teak.

#### 6.1.3 Optimizing intertemporal choice

Our formulation of environmental precarity is inspired by the lived experiences of people from lower socio-economic communities whose future plans are often washed out by unpredictable events like an unforeseen job loss or a massive healthcare bill. Though waiting for the long term is often optimal, the short term can be more beneficial in these precarious conditions. In our farming game, we had posed a similar temporal optimization problem.

Teak provides the maximum crop yield at the end of six trials; thus, choosing teak is the optimal decision in periods of stability. However, during volatile farming conditions, opting for teak would lead one to wait for six trials and accrue heightened farming expenses and crop losses without crop yield. Thus, even though investing in the long-term crop is optimal in terms of stability, it may not be optimal in precarious conditions due to high immediate costs and delayed benefits.

In our experiments in Mitra et al. (2025), we presented this optimization problem to participants in the guise of the farming game. We found that people prefer shorter planning horizons under precarious conditions. This manuscript investigated whether such preference deviations could help us estimate time distortions that account for the observed shift in intertemporal choices. While people may shift their preferences due to perceived shifts in optimality in the paradigm, we tested if time distortion estimates could also explain these shifts, as we think these psychological states (latent shift in optimality and perceived time distortion) can co-occur and affect an individual's choices.

#### 6.1.4 Gameplay walkthrough

**Figure 5:**
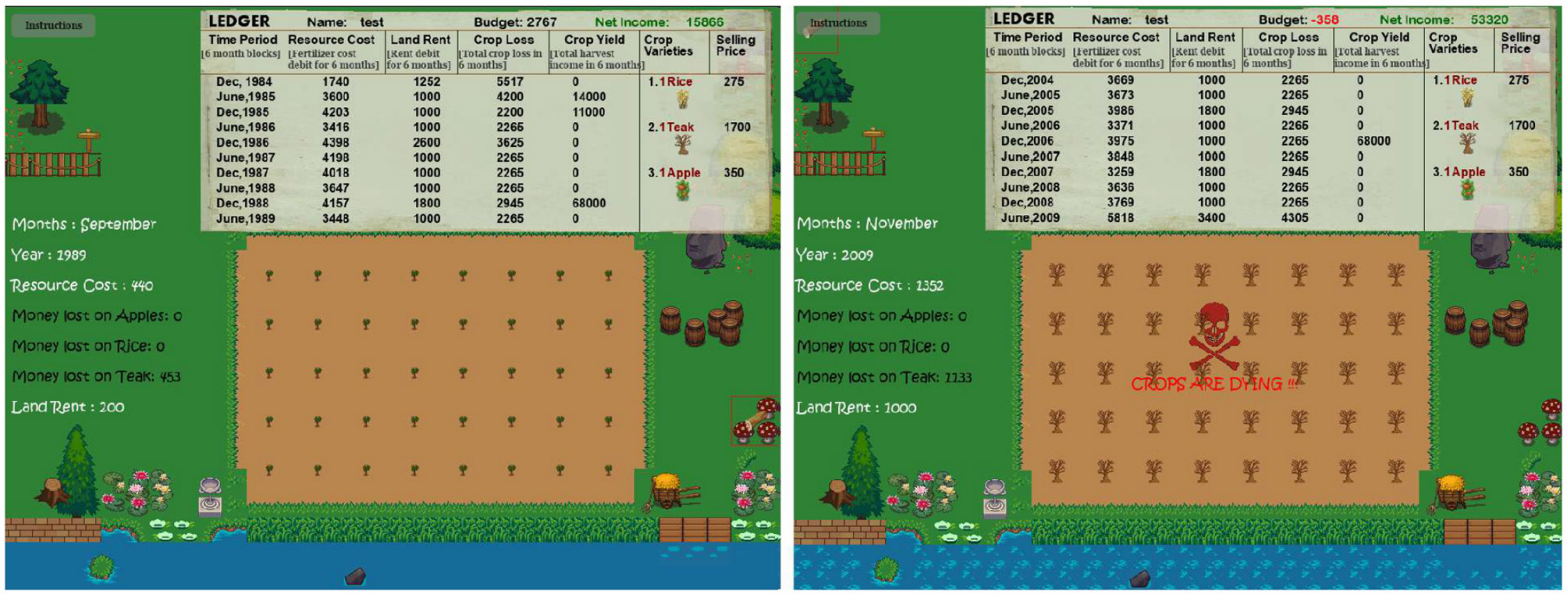
The first and second subplots display the game GUI of our farming experiment in stable and volatile farming conditions. In both images, the top of the GUI shows the “Ledger,” which displays the trial-wise debits and credits of participants for the past ten trials and gets updated in real time. The left portion of the GUI displays the time (month and year), farming expenditures, and crop losses for each crop for the ongoing trial. Cumulative farming expenses, crop losses, and land rents are shown as “Resource Cost,” “Crop Loss,” and “Land Rent” on the Ledger. The middle of the GUI shows the field plot where participants cultivate crops. Participants were notified that crop sowing patterns on the soil or time (month/year) did not affect crop losses or yields. The game can be played online here.

Figure 5 displays the farming game GUI used by our participants. In both experiments, participants played the role of a farmer and were made aware of the game mechanics and how to play it, as well as the choices available in-game using explicit instructions. They were notified that the brown patch of land was the plot for growing crops. A green outline box around the cursor suggested the soil areas available to sow crops, and a red box suggested areas that were not. Sowing the crops on the plot was done with left mouse clicks. Planting each unit of all crops was accompanied by a deduction of 50 coins as the buying price. Choosing which crops to grow (i.e., browsing through options) was done by revolving the mouse wheel. After sowing the plot, participants pressed the Spacebar to start each trial.

**Figure 6:**
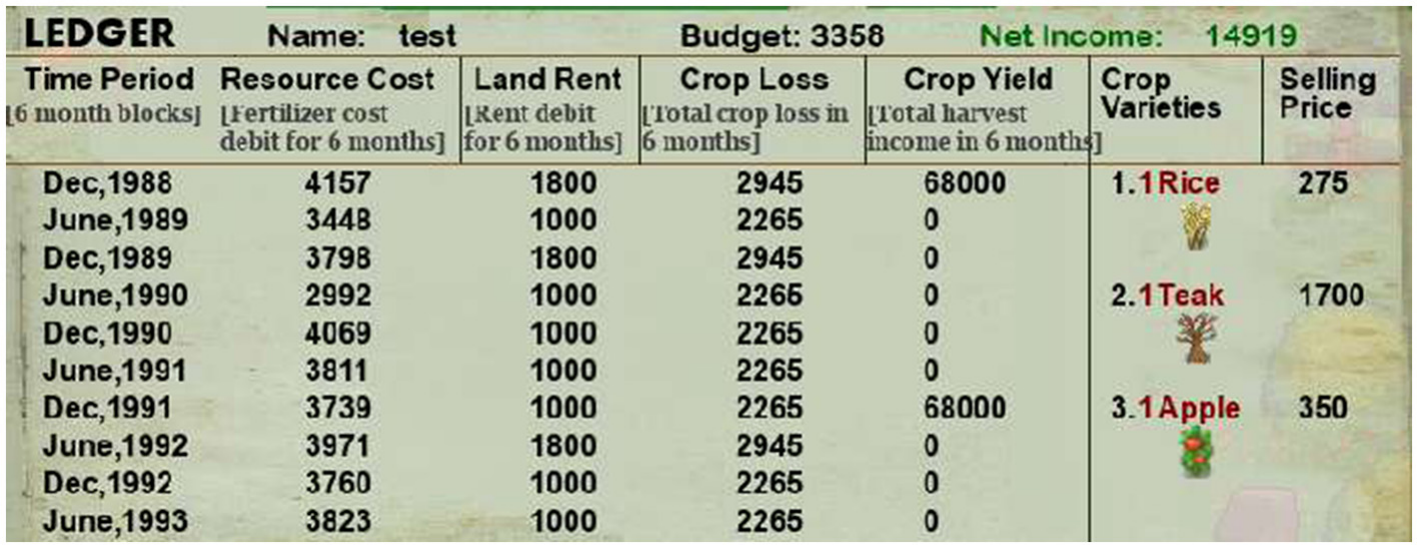
This figure displays the ledger showing farming expenses and crop yield for the past ten trials. It also displays the selling price of each crop type on its right-hand side. The top shows the name, the budget (deducted at the start of every trial), and the net income in the game. Participants were asked to maximize this net income.

Each trial was designed to last six months (15 s in-game). While the trial was ongoing, participants saw the month and year of game-play, corresponding farming expenses deduction from their budget, crop loss, and land rent deductions from their “Net Income,” all updated in real-time (as shown in Figure 6). At the end of each trial, the game paused, and participants had to harvest the full-grown crops (indicated by a sparkling effect) using the right mouse clicks. Crop yield accumulated from the harvest was added to their “Net Income.” After harvesting, participants replanted the plot again and started the subsequent trial. Participants were unaware of the objective duration of each trial, and we asked them to provide their subjective estimates of trial duration without anchoring on the objective duration.

During game-play, participants' crop profit, crop loss, resource cost deductions, and land rent were saved in a ledger for each trial for the past ten trials. They were updated after every trial, and participants were advised to consult these to get a comprehensive view. The ledger also illustrated their net income, budget in hand, and crop profit for each unit of all three crops. Lastly, participants in both experiments were explicitly asked to play to maximize their “Net income.” A ranking-based system was also designed to reward the top three ranks with $25, $16, and $8 in addition to their participation fees of $10 (PPP adjusted).

### 6.2 Analysis protocol and sample details

Since participants' choice of crops was our measure of temporal preferences, we hypothesized that preference for delayed reward would decrease as a result of shocks. Since the yield time for teak was six trials, we identified the preference shift over each shock by taking the average probability of crop choice six trials before and after each resource shock. Mathematically, it can be represented as:


$$\begin{array}{l} \delta p=\frac{\sum_{t+1}^{t+6}p_{a}}{6}-\frac{\sum_{t}^{t-5}p_{b}}{6} \end{array}$$

where *t* is the trial on which resource shock occurs, *p*_*a*_ and *p*_*b*_ are the probabilities of choosing each crop, which were calculated as


$$\begin{array}{l} p_{a}=\frac{n{\left(c\right)}_{a}}{40}\text{and }p_{b}=\frac{n{\left(c\right)}_{b}}{40} \end{array}$$

*c*∈[apples, rice, teak], *n*(*c*)_*b*_ and *n*(*c*)_*a*_ denote the number of respective crops chosen before and after shock within the 40 crop slots on the farmland. Thus, if a participant faced *N* resource shock trials, shifts in preference Δ*p* averaged over all such trials were obtained using


$$\begin{array}{l} \Delta p=\sum \delta p/N \end{array}$$

This Δ*p* provided a participant-level metric of how crop preference shifted as a function of resource shocks in our experiment.

We used the same data as the one published in Mitra et al. (2025) for our analysis in this draft. Our replication study was partly exploratory as we did not have any estimates of how time distortions vary relative to preference deviations. Assuming a low correlation of –0.25, β = 0.80 and α = 0.05 in GPower (Faul et al., 2007), we got a required sample size of 97. We collected data from 102 individuals, of which six participants were identified as outliers using a 1.75*IQR exclusion criterion similar to the one used in Mitra et al. (2025). Our final analysis was done with 96 participants (20 females, *M*_age_ = 21).

### 6.3 Derivation of time distortions from shifts in the discount rate of studies mentioned in Table 1

#### 6.3.1 Haushofer et al. (2013)

In this study, the authors investigated the effects of lab-induced income shocks on discounting. They formulated a laboratory experiment where participants received high and low starting endowments and performed an effortful task to earn money. Among these groups of high and low endowments, some received a positive and negative income shock such that their average incomes in the task were equal. So, an individual in a high-endowment-negative-shock group would have his income equal to that of another in a low-endowment-no-shock group. The authors then tested future discounting using intertemporal choice tasks. They found that the negative-income-shock group exhibited higher discounting or lower indifference points [*M* = 16.43, 95% CI = (13.80, 19.07)] in the intertemporal tasks compared to the always-poor group [*M* = 19.43, 95% CI = (17.02, 21.85)].

For our computation, we used the 95% CI of indifference points reported in the paper for negative and no shock conditions to construct the range where *k*_*a*_ and *k*_*b*_ may lie. Next, we drew *n* random samples of *k*_*a*_ and *k*_*b*_ with replacement from this range (*n* = no. of participants in each group) and obtained the mean shift in discount rates using Δ*k* = *k*_*a*_−*k*_*b*_. Lastly, we obtained the scaled time distortion by dividing Δ*k* by *k*_*b*_. We did this 10,000 times to obtain a bootstrapped distribution of scaled time distortions, giving us the median and 95% CI.

#### 6.3.2 Bickel et al. (2016)

In this study, the authors manipulated income shock to be negative, neutral, and positive, and discount rates were measured across four conditions: future gain, future loss, past gain, and past loss. Furthermore, discounting was measured using intertemporal tasks, which were framed by using the standard method ($X now vs. $X+dx later) or by making the alternate zero explicit ($X now and $0 later vs. $0 now and $X+dx later).

For our calculation, we consider the effects of negative and neutral income shock narratives on the future gain option in the implicit standard framings. Thus, reported discount rates of negative scenarios constituted post-shock conditions, and neutral scenarios were pre-shock conditions in our formulation. The authors calculated changes in discount rate by taking the log transform of *k* in their dataset. Following their footstep, we obtained the log transformed discount rates associated with exposure to neutral and negative income shock narratives by randomly sampling and we find take their difference Δ*ln*(*k*). We derived scaled time distotion from Δ*ln*(*k*) using


$$\begin{array}{l} \;\Delta ln\left(k\right)=ln\left(k_{a}\right)-ln\left(k_{b}\right)\\ \;\Rightarrow \Delta ln\left(k\right)=ln\left(\frac{k_{a}}{k_{b}}\right)\\ \;\Rightarrow \frac{k_{a}}{k_{b}}=exp\left(\Delta ln\left(k\right)\right)\\ \;\Rightarrow \frac{k_{a}}{k_{b}}-1=exp\left(\Delta ln\left(k\right)\right)-1\\ \;\Rightarrow \frac{\Delta k}{k_{b}}=exp\left(\Delta ln\left(k\right)\right)-1\\ \;\Rightarrow \frac{\Delta t}{t_{b}}=exp\left(\Delta ln\left(k\right)\right)-1\;\left[\text{since}\frac{\Delta k}{k_{b}}=\frac{\Delta t}{t_{b}}\right] \end{array}$$

We do this 10,000 times to obtain a bootstrapped distribution of scaled time distortions, which gave us the 50th (median) and its 95% confidence interval by taking the 47.5th percentile above and below the median.

#### 6.3.3 Falco et al. (2019)

In this study, the authors investigated the effects of income variations on discounting among farming households in Ethiopia. They obtained farmers' discount rates using intertemporal tasks like “Would you prefer a payment of 50 Ethiopian Birr today or 65 Ethiopian Birr after 12 months?” and reported a mean discount rate of 0.09 with a standard deviation of 0.04 among 651 farmers.

Income variations were measured as a function of rainfall anomalies, and subjective discount rates among farmers were studied as a function of these income variations. The authors found that farmers preferred smaller immediate rewards when they encountered adverse income shocks—farm income changed as a function of rainfall variations by 0.658 with a standard error of 0.328, and discount rates changed as a function of farm income by –0.0447 with a standard error of 0.0201 while controlling for other idiosyncratic shocks and demographic variables. Using the mean and standard error of regression coefficients mentioned in the study, we constructed a 99% CI range at which their values may lie.

In this study, increased rainfall anomalies reduced farm income, leading to discount rate increases. So, to identify Δ*k*, we randomly sampled from the determined range of regression coefficients, which suggests changes in discount rate per unit decrease in rainfall. Discount rates *k* were sampled using the data from the experiment. In both cases, we obtained *n* samples of Δ*k* and *k* with replacement (*n* = no. of farmers) and derived scaled time distortion by dividing the mean Δ*k* by the mean *k*. We did this 10,000 times to construct a bootstrapped distribution of scaled time distortion. We report the median time distortion and its 95% confidence interval by taking the 47.5th percentile above and below this median.

#### 6.3.4 Blain et al. (2021)

In this study, the authors investigated whether negative income shocks and negative affect brought on by the COVID-19 pandemic affected future discounting and whether they were independent. For our purposes, we were interested in seeing if discount rates changed due to income shocks experienced by individuals during the pandemic. The authors tested 1,145 individuals during the market crash in March 2020 and retested 200 during the market recovery in June 2020. Income shocks were measured using a subjective scale, where participants indicated the impact of COVID-19 on their income on a 6-point scale. Discount rates were measured using Kirby and Petry (2004)'s scale, which made participants choose between a smaller, immediate, and a larger, delayed reward over multiple-choice trials. They found that temporal discounting rates were related to increases in perceived income shocks across individuals by β = 0.14 with a standard error of 0.06 while correcting for demographics.

Thus, for our computations, shifts in discount rates are given by the β parameter, and an individual's shift in discount rate lies in the 99.9% confidence interval of the β parameter. Since discount rates were not reported in the paper, we randomly sampled participants' hypothetical discount parameters from Kirby and Petry (2004)'s scale, where discount parameters vary from 0.00016 to 0.25. Like above, we sampled discount rates and shifts in discount rate *n* times, where *n* = no. of participants to calculate the mean discount rate and shift in discount rate. Then, we constructed a bootstrapped distribution of scaled time distortions by iterating 10,000 times and obtained its median and its 95% CI.

#### 6.3.5 Meles et al. (2024)

In this study, the authors used household panel data from Ethiopia to investigate how risk and time preferences changed due to droughts and rainfall anomalies. Environmental shocks were measured using self-reports of droughts and rainfall shocks calculated as shortfalls relative to the long-term average during the main rainy season. Discounting was measured similarly to Falco et al. (2019)'s study, where participants were asked to choose between 100 Birr today and 100+X Birr a month from today.

Discount rates and shocks were measured in 2010, 2012, and 2014. The authors report that 66%, 68.1%, and 86.6% of the participants chose the immediate reward for 2010, 2012, and 2014 (discount rates > 0.41). 10.9%, 10.6%, and 3.9% participants preferred the sooner option first and switched the delayed reward later (discount rates: from 0.22 to 0.41), and 23.1%, 21.2%, and 9.5% participants always preferred the delayed option (discount rates: 0 to 0.22) in 2010, 2012, and 2014, respectively.

The authors found that the mean preference for delayed reward decreased by α = 0.14 with a standard error of 0.05 as a function of droughts and by β = 0.26 with a standard error of 0.07 as a function of rainfall anomalies. We utilize these preference changes to calculate our variable of interest—time distortions.

We began by simulating the distribution of discount rates (using data from all three waves), which led us to a metric of mean discount rates over time for 4,326 households. Δ*p* was constructed from the reported mean shifts in preference caused by droughts and rainfall shortfalls (α and β). Given these discount rates and measured shift in preference, we calculated *p*_*b*_ and *p*_*a*_ = *p*_*b*_+Δ*p*, using our conceptual framework mentioned in Section 2. Finally, using these *p*_*b*_ and *p*_*a*_, we calculated *t*_*b*_ and *t*_*a*_, which gave us scaled time distortions for our sample of 4,326 households. We repeat this process 1,000 times to estimate its bootstrapped distribution and report its median and 95% confidence intervals separately for droughts and rainfall anomalies.

## Data Availability

The datasets presented in this study can be found in online repositories (https://osf.io/q4tdh/?view_only=f6d564abeed84981bffd958e074d8597). The names of the repository/repositories and accession number(s) can be found in the article/supplementary material.

## Footnotes

1 We defined precarity as any exogenous and unpredictable event that may bring forth a state of uncertainty in an agent's immediate environment, leading to changes in their mental and physical states (i.e., thoughts and behaviors). While we have operationalized precarity as heightened expenditures and losses in the game, it can also include non-economic exogenous perturbations such as health risks.

2 To do these computations, we assumed the ratio of sooner and delayed rewards to equal the ratio of profits associated with the game's short-term and long-term crop choices. Thus, the sooner reward *r*_0_ was equal to 1, the delayed reward *r*_*t*_ was equal to 6.3, and the delay *t*_*b*_ was equal to 6 (ratio of the objective delay between fruition of teak and apple/rice).
